# Antioomycete Nanoformulation for Biocontrol of English Walnut Crown and Root Rot Caused by *Phytophthora cinnamomi*

**DOI:** 10.3390/plants14020257

**Published:** 2025-01-17

**Authors:** Aldo Salinas, Iván Montenegro, Yusser Olguín, Natalia Riquelme, Diyanira Castillo-Novales, Alejandra Larach, Laureano Alvarado, Guillermo Bravo, Alejandro Madrid, Juan E. Álvaro, Ximena Besoain

**Affiliations:** 1 Laboratorio de Fitopatología, Escuela de Agronomía, Facultad de Ciencias Agronómicas y de los Alimentos, Pontificia Universidad Católica de Valparaíso, Casilla 4-D, Quillota 2260000, Chile; aldo.salinas@pucv.cl (A.S.); natalia.riquelme@pucv.cl (N.R.); diyaniracastillonovales@gmail.com (D.C.-N.); alejandra.larach@pucv.cl (A.L.); l.alvarado.borella1@gmail.com (L.A.); juan-eugenio.alvaro@pucv.cl (J.E.Á.); 2 Center of Interdisciplinary Biomedical and Engineering Research for Health (MEDING), Escuela de Obstetricia y Puericultura, Facultad de Medicina, Universidad de Valparaíso, Angamos 655, Reñaca, Viña del Mar 2520000, Chile; bravoc.guillermo@gmail.com; 3 Millennium Nucleus Bioproducts, Genomics and Environmental Microbiology (BioGEM), Avenida España 1680, Valparaíso 2390123, Chile; alejandro.madrid@upla.cl; 4 Departamento de Química y Medio Ambiente, Universidad Técnica Federico Santa María, Avenida España 1680, Valparaíso 2390123, Chile; yusser.olguin@usm.cl; 5 Centro Científico y Tecnológico de Valparaíso (CCTVal), Universidad Técnica Federico Santa María, Avenida España 1680, Valparaíso 2390123, Chile; 6 Centro de Biotecnología, Universidad Técnica Federico Santa María, Avenida España 1680, Valparaíso 390123, Chile; 7 Laboratorio de Microbiología Molecular y Biotecnología Ambiental, Departamento de Química & Centro de Biotecnología Dr. Daniel Alkalay Lowitt, Universidad Técnica Federico Santa María, Avenida España 1680, Valparaíso 2390123, Chile; 8 Laboratorio de Productos Naturales y Síntesis Orgánica (LPNSO), Departamento de Ciencias y Geografía, Facultad de Ciencias Naturales y Exactas, Universidad de Playa Ancha, Avda. Leopoldo Carvallo 270, Playa Ancha, Valparaíso 2360004, Chile

**Keywords:** *Phytophthora cinnamomi*, crown and root rot, plant extract, nanoemulsions, English walnut

## Abstract

In Chile and worldwide, walnut (*Juglans regia L.*) production faces significant losses due to crown and root rot caused by the phytopathogen *Phytophthora cinnamomi*. Currently, control methods have proven insufficient or unfavorable for the environment, increasing the need for sustainable alternatives. This research evaluates nanoemulsions based on extracts of medicinal plants endemic to Chile to control *P. cinnamomi* in walnut crops. The methodology included an in vitro test to determine the effective inhibitory concentrations of three nanoemulsions (N80, N90, and N100) on the mycelial growth of the phytopathogen, a test on walnut plants under controlled conditions, and two field tests using concentrations between 300 and 500 ppm. The in vitro results showed that the nanoemulsions could inhibit 90% of mycelial growth at 80 to 100 ppm concentrations. In the field, the N90 nanoemulsion at 500 ppm significantly reduced disease symptoms preventively and post-inoculation, compared with the control. This research is the first to study the use of nanoemulsions from native Chilean plants to control *P. cinnamomi*, showing potential to reduce the use of synthetic fungicides, contributing to safer and more ecological phytosanitary management.

## 1. Introduction

The English walnut (*Juglans regia* L.) is a fruit tree of global importance. According to data from the Food and Agriculture Organization of the United Nations (FAO), in 2022, walnut plantations spanned a global area of 1,247,938 hectares, producing approximately 3.8 million tons of in-shell nuts. Asia has emerged as the largest producer, contributing 56.7% of global production [1]. In Chile, walnut plantations covered 44,626 hectares in 2023 and 11.96% of the country’s commercial fruit tree area. These data place walnuts as the most widely cultivated fruit trees in Chile, surpassed only by cherry [2].

Despite its economic significance, the walnut tree faces numerous challenges, particularly diseases caused by bacteria, fungi, and oomycetes. These pathogens can severely affect the commercial quality of nuts and cause significant yield losses. Among the most notable phytopathogens are fungi from the *Botryosphaereacea* and *Diaporthaceae* families [3,4], bacteria of the genus *Xanthomonas*, and oomycetes of the genus *Phytophthora*, which cause diseases such as crown and root rot [5,6]; this last disease is primarily due to a polyphagous microorganism known as *Phytophthora cinnamomi*. This pathogen infects more than 5000 plant species [7,8,9] and primarily gains entry through feeder roots [10,11,12].

In Europe, *P. cinnamomi* is recognized as one of the most aggressive species, causing decline, and crown and root rot in walnut trees in countries like France and Italy [13,14]. In Chile, this disease can lead to yield losses of up to 12% [15]. Crown and root rot is caused by a soilborne pathogen propagated by zoospores, which infects plants through the encystment of these spores in the feeder roots [16]. The pathogen can also survive in soil or plant debris chlamydospores or grow saprophytically on decomposing organic matter [17]. Symptoms of disease caused in walnut trees include reduced sprouting, leaf chlorosis, decreased crown density, defoliation, cortical root rot, basal trunk rot with dark exudates, and, in severe cases, tree death [6,18,19].

Management of *Phytophthora* relies heavily on preventive strategies. Recommended practices include planting in well-drained soils, acquiring healthy plants, using resistant or tolerant rootstocks, monitoring for soilborne pathogens, managing irrigation appropriately, and applying balanced nitrogen fertilization [20,21]. Soil preparation techniques, such as subsoiling of the soil to break compact soil layers are also effective [10]. Chemical fungicides like potassium phosphite, metalaxyl, and fosetyl-Al are commonly used for disease control [16]. However, tolerance and reduced sensitivity have emerged in some *P. cinnamomi* isolates [22,23] highlighting the need for alternative strategies to minimize reliance on synthetic chemicals.

Resistant clonal rootstocks (e.g., Vlach, VX211, and RX1) and biological control present promising alternatives. Resistant rootstocks activate defense mechanisms involving sugars and phenols to counter infection by *P. cinnamomi* and *P. citrophthora* [24,25]. However, in Chile, most walnut trees are grafted onto *J. regia* rootstock, which is highly susceptible to *P. cinnamomi*. Biological control agents like *Trichoderma* and *Bacillus* species have also shown potential for managing *Phytophthora* [26]. Growing environmental and food safety concerns have spurred interest in natural crop protection agents, focusing on plant extracts and their secondary metabolites [27]. Medicinal plant extracts are a viable alternative, offering antifungal properties without environmental or health risks. Nanotechnology further enhances these approaches, enabling the development of innovative tools for effective pathogen control [28].

Nanotechnology represents a significant opportunity for sustainable agricultural solutions. The global nanoemulsions market, valued at USD 6.8 billion in 2017, is projected to grow at an annual rate of 9%. Regions such as Europe, Asia-Pacific, and North America are at the forefront of adopting nanopesticide technologies [29,30]. Nanoparticles offer unique properties, such as enhanced reactivity and improved product dispersion, due to their high surface-to-volume ratio [31,32]. Nanoformulations reduce active compound doses, minimize environmental contamination, and enhance solubility uniformity during application [33].

A sustainable strategy for developing nanofungicides involves combining nanotechnology with plant bioactive compounds. For example, endemic Chilean plants like *Psoralea glandulosa* (culén), a resinous shrub used as an antiseptic to treat infections caused by fungi [34], and *Escallonia illinita* (barraco), a resinous aromatic shrub used in liver diseases. These plants contain compounds such as meroterpene and pinocembrin, demonstrating antifungal and antibacterial activities [35,36,37].

Nanoemulsions formulated from *P. glandulosa* and *E. illinita* exhibit antifungal properties primarily due to their main metabolites, bakuchiol and pinocembrin. These compounds act by disrupting the integrity of the pathogen’s cell membrane, leading to the leakage of essential cellular contents and, ultimately, fungal death [36,38]. Additionally, pinocembrin can inhibit key enzymes required for the pathogen’s growth and proliferation, while bakuchiol has demonstrated a direct activity against various fungal and oomycete isolates [39]. These properties, enhanced by the nanoemulsion’s ability to improve the stability and bioavailability of the active compounds, suggests a synergistic mechanism of action effective against *P. cinnamomi*, making them a promising tool for sustainable plant disease management.

Considering the above background, the present study explores some ecological and sustainable alternatives to chemical fungicides for managing *P. cinnamomi*. Therefore, this research evaluates the efficacy of nanoemulsions derived from the plant exudates of *Psoralea glandulosa* and *Escallonia illinita* for controlling *Phytophthora cinnamomi* in vitro, and the disease it causes in English walnut plants under controlled greenhouse and experimental field conditions.

## 2. Results

### 2.1. In Vitro Trial of Nanoemulsions for the Inhibition of P. cinnamomi Mycelium

**Figure 1:**
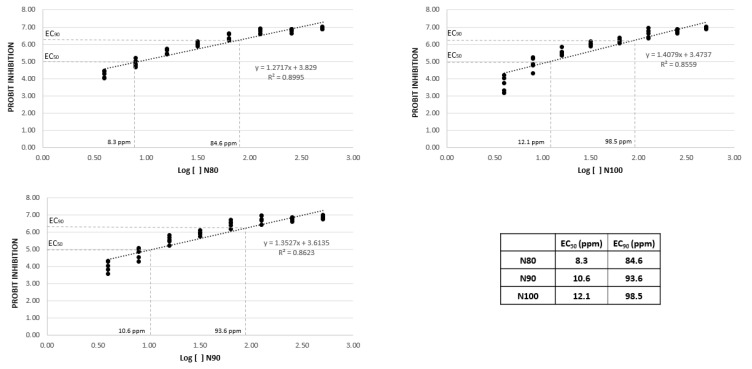
Effect of the concentration of nanoemulsions N80, N90, and N100 (Log []) on the inhibition of micellar growth of *P. cinnamomi* (Probit inhibition), in vitro, on CMA culture medium, 72 h after the pathogen was sown in the medium. Probit inhibition values above 5 and 6.28 correspond to 50% and 90% inhibition of the pathogen mycelium, respectively.

From the in vitro tests carried out with the nanoemulsions N80, N90, and N100 against *P. cinnamomi* (at different increasing concentrations between 1 and 1024 ppm, plus no nanoemulsion), it was determined that the three nanoemulsions used presented an EC50 close to 10 ppm and, with doses between 80 and 100 ppm, they presented inhibitions over 90% of the growth of the oomycete mycelium, observing lower EC50 and EC90 concentrations with the nanoemulsions N80 and N90 (Figure 1). 

### 2.2. Greenhouse Trial of Nanoemulsions in Plants Inoculated with P. cinnamomi

**Figure 2:**
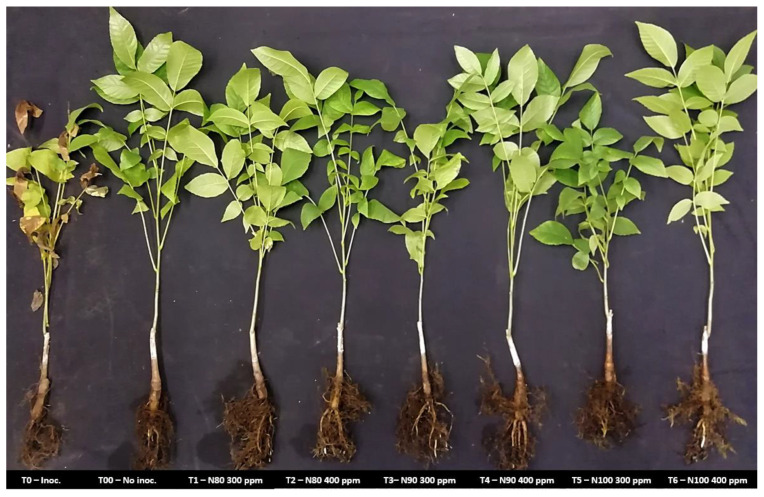
Effect of the use of nanoemulsions on 12-month-old Chandler walnut plants on English walnut *(J. regia*) rootstock. From left to right: (T0) Control inoculated with *P. cinnamomi* and not treated with nanoemulsions; (T00) Control not inoculated with *P. cinnamomi* and not treated with nanoemulsions; (T1) Plants treated with N80 at 300 ppm and inoculated; (T2) Plants treated with N80 at 400 ppm and inoculated; (T3) Plants treated with N90 at 300 ppm and inoculated; (T4) Plants treated with N90 at 400 ppm and inoculated; (T5) Plants treated with N100 at 300 ppm and inoculated; (T6) Plants treated with N100 at 400 ppm and inoculated. Photograph taken 15 days after inoculation of the phytopathogen.

**Figure 3:**
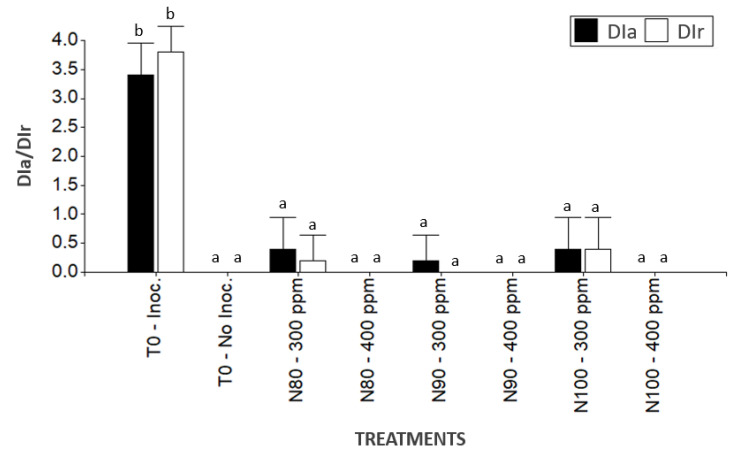
Aerial and root damage index of walnut plants (*J. regia*) inoculated, treated, and untreated with nanoemulsions and not inoculated with *P. cinnamomi*. Separation of means with Kruskal-Wallis test. Means with different letters indicate significant differences from each other (*p* ≤ 0.05).

In the greenhouse trial conducted on the walnut plants grown in substrate, it was observed that 14 days after inoculation and treatment application, the inoculated plants without nanoemulsions treatments exhibited symptoms associated with the disease, including 75–100% foliar damage and root rot (Figure 2). In contrast, the plants treated with nanoemulsions N80, N90, and N100 at concentrations of 300 and 400 ppm, despite being inoculated with the phytopathogen, showed no visible damage. These treated plants did not present significant differences compared with the non-inoculated or untreated control plants. Moreover, no symptoms of phytotoxicity were observed in any of the plants treated with the nanoemulsions (Figure 3).

### 2.3. Field Trial of Nanoemulsions on Plants Inoculated with P. cinnamomi

**Figure 4:**
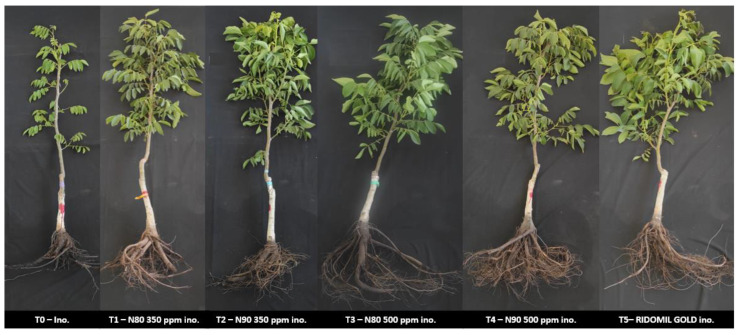
Effect of N80 and N90 nanoemulsions on English walnut (*J. regia*) rootstock plants. From left to right: (T0) Control inoculated with *P. cinnamomi* and not treated with nanoemulsions; (T1) Plants treated with N80 at 350 ppm and inoculated; (T2) Plants treated with N90 at 350 ppm and inoculated; (T3) Plants treated with N80 at 500 ppm and inoculated; (T4) Plants treated with N90 at 500 ppm and inoculated; (T5) Plants treated with Ridomil Gold and inoculated.

From the preventive test on the English walnuts planted in soil with ridges in the field, the plants were inoculated at 350 ppm and 500 ppm with the N80 and N90 nanoemulsions. Symptoms associated with the disease were observed in the inoculated plants and in the plants without nanoemulsion treatment. Unlike the other treatments carried out, the plants affected by the phytopathogen showed a noticeable decrease in rootlets and root rot. In the aerial part, lower canopy development and defoliation were observed (Figure 4).

**Figure 5:**
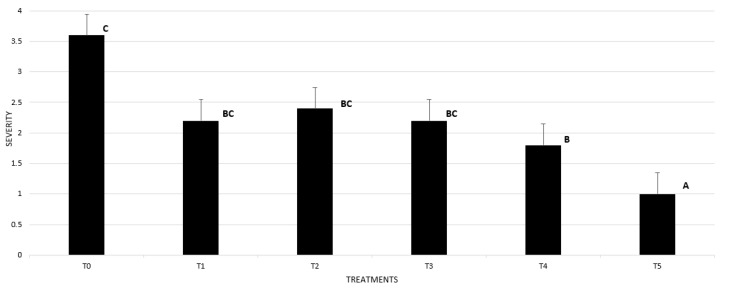
Severity of symptoms in walnut (*J. regia*) roots inoculated, and not inoculated with *P. cinnamomi* treated with nanoemulsions: (T0) Control inoculated with *P. cinnamomi* and not treated with nanoemulsions; (T1) Plants with preventive treatment with N80 at 350 ppm and inoculated; (T2) Plants with preventive treatment with N90 at 350 ppm and inoculated; (T3) Plants with preventive treatment with N80 at 500 ppm and inoculated; (T4) Plants with preventive treatment with N90 at 500 ppm and inoculated; (T5) Plants with preventive treatment with Ridomil Gold and inoculated. Separation of means with Kruskal-Wallis test. Means with different letters indicate significant differences from each other (*p* ≤ 0.05).

Likewise, the plants in the treatment inoculated with *Phytophthora cinnamomi* and with the application of the N90 nanoemulsion at 500 ppm, together with the chemical treatment, were the only treatments that presented a significantly lower disease severity than the inoculated treatment without the application of nanoemulsions. No symptoms of phytotoxicity were observed from use of the nanoemulsions (Figure 5).

### 2.4. Preventive and Post-Inoculation Field Trial with N90 Nanoemulsion on Walnut Plants

**Figure 6:**
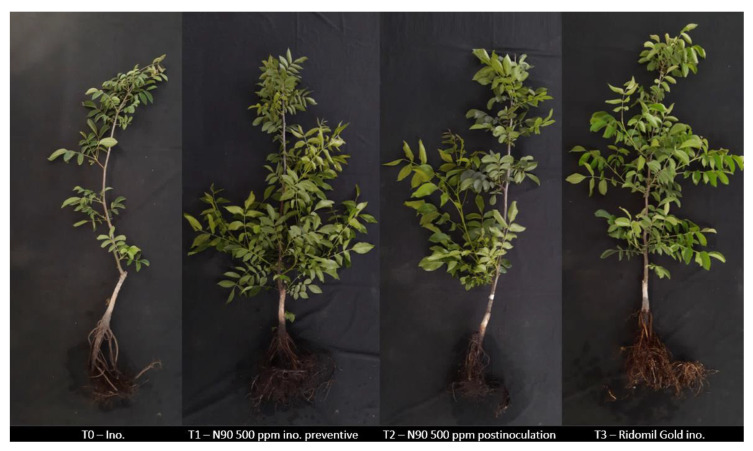
Effect of N90 nanoemulsion on English walnut (*J. regia*) rootstock plants. From left to right: (T0) Control inoculated with *P. cinnamomi* and not treated with nanoemulsions; (T1) Plants with preventive treatment with N90 at 500 ppm and inoculated; (T2) Plants with post-inoculation treatment with N90 at 500 ppm and inoculated; (T3) Plants with preventive treatment with Ridomil Gold and inoculated.

**Figure 7:**
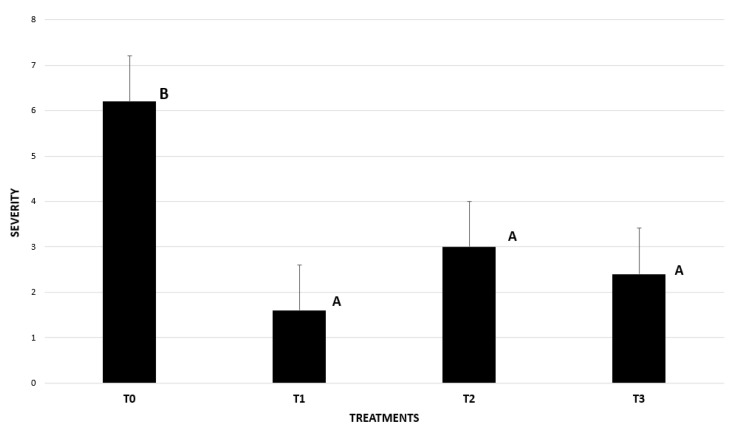
Severity of symptoms in walnut (*J. regia*) roots inoculated and not inoculated with *P. cinnamomi* treated with nanoemulsions: (T0) Control inoculated with *P. cinnamomi* and not treated with nanoemulsions; (T1) Plants with preventive treatment with N90 at 500 ppm and inoculated; (T2) Plants with post-inoculation treatment with N90 at 500 ppm and inoculated; (T3) Plants with preventive treatment with Ridomil Gold and inoculated. Separation of means with Kruskal-Wallis test. Means with different letters indicate significant differences from each other (*p* ≤ 0.05).

In the preventive and post-inoculation trials on the English walnut plants in soil, the plants were inoculated at 500 ppm with the N90 nanoemulsion. Symptoms associated with the disease were observed on the inoculated plants and on the plants without nanoemulsion treatment. Unlike the other treatments, the plants affected by the disease showed a notable decrease in the number of rootlets and root rot. In the aerial part, lower canopy development and defoliation were observed (Figure 6). In addition, all treatments presented a significantly lower disease severity than the inoculated treatment without applying nanoemulsions. No symptoms of phytotoxicity were observed from use of the nanoemulsions (Figure 7).

## 3. Discussion

Plant extracts, such as those utilized in this study, present a viable alternative to chemical pesticides, effectively controlling phytopathogenic microorganisms without adversely impacting human health or the environment [40]. Compounds derived from natural products, such as 2′-hydroxy-chalcones, also show promise against oomycetes, addressing the need to reduce the negative side effects of synthetic fungicides, including chemical residues [41]. Previous research has evaluated plant extracts for their effectiveness in controlling *P. cinnamomi.* For instance, oregano (*Lippia berlandieri*) extracts achieved EC_50_ and EC_100_ values of 60 ppm and 108.3 ppm [42], respectively. Similarly, Andrade-Hoyos et al. [43] reported a total mycelial inhibition at 120 ppm using clove (*Syzygium aromaticum*) extracts. In this study, the nanoemulsions derived from *Escallonia illinita* and *Psoralea glandulosa* achieved significantly lower EC_50_ values (approximately 10 ppm) and EC_90_ values (80–100 ppm), underscoring their enhanced potency through nanoencapsulation.

These results align with the findings by Montenegro et al. [36]; they observed a “Minimum Inhibitory Concentration (MIC)” of 75 ppm when using a resinous extract of *E. illinita* against oomycetes of the genus *Saprolegnia*. Similarly, Madrid et al. [35], using an extract of *P. gladulosum*, an inhibition of up to 80% of the oomycete *P. cinnamomi* was observed using 150 ppm. These studies, combined with the findings presented here, highlight the ability of nanoemulsions to enhance the efficacy of plant extracts, offering a promising avenue for sustainable agricultural practices. Furthermore, nanoemulsions have shown potential in broader applications, such as controlling *Fusarium oxysporum* in tomato plants, with no observed damage in treated plants; these findings are consistent with this study where walnut plants treated with nanoemulsions at 300 and 400 ppm also exhibited no symptoms of damage [44].

The results obtained reinforce the idea that nanoemulsions formulated with bakuchiol and pinocembrin might control *P. cinnamomi* through complementary mechanisms. Bakuchiol, derived from *Psoralea glandulosa*, disrupts the pathogen’s cell membrane causing a loss of cellular integrity and the inhibition of essential metabolic processes of unicellular fungi [39]. Meanwhile, pinocembrin, found in the resinous exudate of *Escallonia illinita*, inhibits critical enzymatic activity and prevents the development of structures such as zoospores and germ tubes [37,39]. These actions are further enhanced by the physical properties of nanoemulsions, such as their nanometric size which increases the surface area for interaction and improves penetration into the pathogen cells. Additional studies should explore whether these mechanisms also enable a direct interaction with plant tissues, further enhancing their effectiveness in early infection stages.

The incorporation of nanoemulsions into agricultural systems offers significant environmental and economic benefits. Nanoemulsions reduce the chemical residue loads in soil and water, leveraging natural antifungal compounds to minimize toxic effects on non-target organisms and preserve biodiversity. Matei et al. [45] demonstrated that silver nanoparticle-polyphenol composites effectively inhibit *P. cinnamomi,* providing a framework for eco-friendly crop protection solutions. Similarly, the nanoemulsions tested in this study achieved high efficacy at low concentrations, further mitigating potential environmental contamination. From an economic perspective, the ability to achieve effective control at low doses translates to reduced input costs for farmers, while improved disease management enhances crop yield and quality [1]. The scalability of nanoemulsion production, using existing agricultural and chemical manufacturing infrastructure, further supports their adoption in large-scale farming operations. Studies such as those by Du et al. [46], which highlighted the enhanced stability of nanoemulsions in microencapsulated forms, reinforce their potential for widespread agricultural use.

The results of this study demonstrate that nanoemulsions derived from *P. glandulosa* and *E. illinita* represent a significant advancement in sustainable pest and disease management. By incorporating these nanoemulsions into Integrated Pest Management (IPM) strategies, their use can complement existing biological control agents, such as *Trichoderma* and *Bacillus* species, creating a multi-pronged approach to disease suppression [4,47]. Moreover, by reducing the reliance on synthetic fungicides, nanoemulsions can help to mitigate the emergence of fungicide-resistant pathogen strains, a pressing concern in modern agriculture. The demonstrated efficacy of nanoemulsions against *P. cinnamomi* in walnuts suggests their broader application for other crops affected by *Phytophthora* spp., such as avocados, citrus, and ornamentals. Expanding the use of these technologies could enhance agricultural resilience to soilborne diseases, particularly in regions heavily affected by these pathogens.

Although fungicides are often considered as biological preventive measures, the results obtained in this study agree with those of Heflish et al. [48], who demonstrated that a plant-derived fungicide derived from *Plantago lagopus* could effectively control *Rhizoctonia solani* in tomato plants, both preventively and post-inoculation. The double efficacy observed in the present study with the N90 nanoemulsion at 500 ppm, controlling *P. cinnamomi* in the walnut plants both preventively and post-inoculation, underlines its versatility and effectiveness. These findings reinforce the ability of *P. glandulosa* and *E. illinita* nanoemulsions to control oomycetes, as previously observed by Madrid et al. [35,38] and Montenegro et al. [36] These studies compared the potential of nanoemulsions with commercial antibiotics, confirming their role as innovative tools in sustainable agriculture. By providing effective and environmentally friendly solutions for disease control, nanoemulsions can contribute significantly to addressing global challenges in agricultural sustainability and food security. As a continuation of this work, ongoing research aims to evaluate the efficacy of these nanoemulsions under different soil conditions to determine if their effects remain consistent across varying environmental factors. This next stage will provide deeper insights into their practical applicability and scalability in diverse agricultural systems.

## 4. Materials and Methods

### 4.1. In Vitro Trial of Nanoemulsions in Inhibiting P. cinammomi Mycelium

The in vitro trial was implemented at the Laboratorio de Fitopatología of the Pontificia Universidad Católica de Valparaíso (PUCV), Escuela de Agronomía located in La Palma, Quillota, Región de Valparaíso, Chile. The nanoemulsions used in the study were provided [36,37] by Dr. Iván Montenegro. These nanoemulsions had previously been evaluated in another study where it was demonstrated that they are non-toxic to plants.

The nanoemulsions (N80, N90, and N100) were prepared as described by Aravena et al. [44], and were evaluated in vitro with the phytopathogenic oomycete *P. cinnamomi* 1955 (GenBank codes: ITS MH236245, β-tubulin MH427884 and COI MH448656) with a previously tested pathogenicity [49], and different concentrations of the nanoemulsions varying between 1 and 1024 ppm (with five repetitions for each concentration) to determine the values corresponding to the effective concentrations (EC_50_ and EC_90_) which controlled the pathogen in Corn Meal Agar (CMA) culture medium.

The CMA culture medium was prepared by dissolving 17 g of the medium in 1 L of distilled water and then autoclaving it at a temperature of 118 °C to 121 °C for 21 min. Then, when the temperature of the culture medium dropped to about 45 °C, 100 uL of the nanoemulsions were incorporated at the desired concentrations, and 20 mL of the culture medium was poured into 90 mm Petri dishes. Forty-eight hours after preparing the petri dishes, the plant pathogen *P. cinnamomi* discs were placed in the center and incubated at 22 °C.

After 72 h of incubation, the equatorial and polar diameters of the oomycete growth were measured. The trial was repeated twice to ensure consistency. Subsequently, a linear regression analysis was performed. The concentration values were transformed into their natural logarithms, and the inhibition percentages were converted into Probit values. The corresponding values for the inhibition percentages were obtained from Finney’s table (Finney, [50]). Using the regression analysis, the EC50 and EC90 values for each nanoemulsion were calculated.

### 4.2. Greenhouse Trial of Nanoemulsions in Plants Inoculated with P. cinnamomi

The in vitro trial findings informed the design of an assay conducted under controlled conditions in a polycarbonate greenhouse maintained at 25 °C, with optimal relative humidity to minimize variability due to environmental factors. Nanoemulsions (N80, N90, and N100) were evaluated, their preparation as described in Aravena et al. [44], briefly: the preparation was made using high-energy methods, including sonication and high-pressure homogenization. The formulation components were selected based on the compatibility of the plant extracts with various oils (coconut, palm, peanut, and soybean oil) and surfactants (e.g., Triton X-100, Triton X-114, (Thermo Fisher Scientific, Waltham, MA, USA, catalog no. BP151) Tween 20, Tween 40, and Tween 80 (Sigma-Aldrich, St. Louis, MO, USA, catalog no. P1379), guided by the hydrophilic-lipophilic balance (HLB) system for o/w emulsions. Further, the extracts and oils were mixed in equal parts, stirred for 48 h, centrifuged, and then filtered. The resulting oil mixture was combined with an aqueous surfactant solution (1%, 3%, or 5%) and processed with a sonicator for 10 min, followed by high-pressure homogenization for 2 h. The nanoemulsion’s transmittance was evaluated via turbidimetry, and the particle size was measured using Dynamic Light Scattering (DLS). Their stability was assessed by monitoring the particle sizes after three heating-cooling cycles (4–40 °C). The most stable formulation was the coconut oil extract with 5% Triton X-100, achieving particle sizes of below 230 nm.

The nanoemulsions were applied at two concentrations to the 6-month-old English walnut (*J. regia* L.) rootstock plants grown from seed and grafted with the Chandler variety. The plants were cultivated in 5-L growth containers filled with a substrate composed of 30% sand, 50% agricultural soil, and 20% peat moss, ensuring an appropriate growing medium. Irrigation was delivered via a technified drip system, controlled by irrigation programmers that regulated intervals and duration. Controlled-release NPK granular fertilizer (Basacote^®^ 6M, Compo Expert, Münster, Germany) was applied according to the recommended doses for walnut plants.

For the trial, 30 mL of each nanoemulsion was applied at concentrations of 300 and 400 ppm, with the treatments compared with negative controls (non-inoculated and untreated) and an inoculated but untreated control group. Twenty-four hours after treatment application, the *P. cinnamomi* inoculum, previously tested for pathogenicity [49], was prepared at a concentration of 1 × 10^4^ zoospores/mL using established protocols [6,49].

The inoculum preparation involved transferring the mycelial discs of *P. cinnamomi* from the CMA medium Petri plates into containers with a carrot juice solution. After 72 h of incubation at 22 °C, the mycelium was separated, briefly placed in a saline solution for 3 min, and then transferred into sterile distilled water. Sporangia in the sterile distilled water were confirmed after 72 h. The sporangia-containing solution was subjected to cold stress to induce zoospore release (the mycelia was filtered with a sterile gauze), and the concentration of zoospores was measured using a Neubauer chamber (Hisrchmann Lab, Eberstadt, Germany).

Following preparation, 30 mL of the inoculum was applied to the corresponding plants, and saturation with irrigation water was performed for 24 h. Fourteen days post-inoculation (when the control plants displayed symptoms of damage due to the pathogen), the aerial and root damage indices were assessed using scales described by Vettraino et al. [14]. The fresh and dry weights of both the aerial parts and roots were also recorded.

The experiment was designed as a completely randomized design (CRD) with eight treatments and five plants per treatment as experimental units. A statistical analysis of the variables was performed using the Kruskal-Wallis test (*p* ≤ 0.05), followed by the Conover-Iman test for pairwise comparisons using the InfoStat software, version 2020.

### 4.3. Field Trial of Nanoemulsions on Plants Inoculated with P. cinnamomi

The preventive trial was conducted at the Phytopathology Laboratory of the Pontificia Universidad Católica de Valparaíso, Escuela de Agronomía, located in La Palma, Quillota, Valparaíso Region, Chile. The trial took place during spring-summer weather conditions, with temperatures ranging from 20 °C to 34 °C, which are optimal for the development of *P. cinnamomi*. The study was carried out on two-year-old walnut plants of the Chandler variety grafted onto *J. regia* rootstocks, planted in clay loam soil within an experimental plot at the Escuela de Agronomía, PUCV.

The trial included six preventive treatments: T0 (inoculated without nanoemulsion application), T1 (N80 at 350 ppm and inoculated), T2 (N90 at 350 ppm and inoculated), T3 (N80 at 500 ppm and inoculated), T4 (N90 at 500 ppm and inoculated), and T5 (Ridomil Gold 480 SL (Syngenta, Basel, Switzerland) and inoculated). Three months after planting, the first application of treatments were performed, with a second application following 15 days later. Each treatment involved the application of 300 mL of nanoemulsion, Ridomil Gold, or water (depending on the treatment).

The inoculum was prepared using a mixture of *P. cinnamomi* isolates (PN 1858, PN 1955, and PN 1956), which had been previously tested for pathogenicity, at a concentration of 1 × 10^4^ zoospores/mL using established protocols [6,49]. Three days after the final application of treatments, 300 mL of the inoculum were applied to the corresponding plants during the first root growth flush in November. Following inoculation, the soil was saturated with irrigation water for 24 h to promote infection.

Three months after inoculation, the effects of the treatments were evaluated based on disease severity (using the damage scale described by Vettraino et al. [14]), the fresh weight of aerial parts and roots, and the number of leaves. The experiment was conducted in an experimental plot using a completely randomized design (CRD), with six treatments, five replications, and one plant per experimental unit. Variables were analyzed using the Kruskal-Wallis test (*p* ≤ 0.05), followed by the Conover-Iman test for pairwise comparisons using the statistical software InfoStat, version 2020.

### 4.4. Preventive and Post-Inoculation Field Trial with N90 Nanoemulsion on Walnut Plants

The trial was conducted at the Phytopathology Laboratory of the Pontificia Universidad Católica de Valparaíso, Escuela de Agronomía, located in La Palma, Quillota, Valparaíso Region, Chile. The study took place during spring-summer weather conditions, with temperatures ranging from 20 °C to 34 °C, which are optimal for the development of *P. cinnamomi*. It was carried out on two-year-old walnut plants of the Chandler variety grafted onto *J. regia* rootstocks, planted in clay loam soil within an experimental plot at the Escuela de Agronomía, PUCV.

The trial included four treatments: T0 (inoculated without nanoemulsion application), T1 (preventive application of N90 at 500 ppm and inoculated), T2 (post-inoculation application of N90 at 500 ppm and inoculated), and T3 (preventive application of Ridomil Gold 480 SL and inoculated). Three months after planting, the applications for the preventive treatments began. Two applications of N90 or Ridomil Gold were performed, with a 15-day interval between the applications. Three days after the final preventive application, the inoculum was prepared using a mixture of *P. cinnamomi* isolates (PN 1858, PN 1955, and PN 1956) with previously tested pathogenicity, at a concentration of 1 × 10^4^ zoospores/mL using established protocols [6,49]. Subsequently, 300 mL of the inoculum was applied to the corresponding plants in the control, preventive, and post-inoculation treatments.

After inoculation, the soil was saturated with irrigation water for 24 h, coinciding with the second root growth flush of the walnut plants in February. Three days post-inoculation, the applications for the post-inoculation treatment began. This involved two applications of the N90 nanoemulsion, also with a 15-day interval between the treatments.

Three months after inoculation, the effects of the treatments were evaluated. Variables included disease severity (assessed using the modified damage scale described by Todd and Kommedahl [51]), the fresh weight of aerial parts and roots, and the number of leaves. The experiment was conducted in an experimental plot using a completely randomized design (CRD), with four treatments, five replications, and one plant per experimental unit. Data were analyzed using the Kruskal-Wallis test (*p* ≤ 0.05), followed by the Conover-Iman test for pairwise comparisons using the statistical software InfoStat, version 2020.

## 5. Conclusions

This study represents the first report in Chile using nanoemulsions formulated from the endemic and medicinal plants *P. glandulosa* and *E. illinita* to control crown and root rot caused by *P. cinnamomi* in English walnut plants. The nanoemulsions demonstrated significant inhibition of oomycete mycelial growth and effectively reduced disease symptoms under controlled conditions. The N90 nanoemulsion showed superior effectiveness compared with N80 and N100 in controlling *P. cinnamomi*, with no significant differences observed when compared with the treatment with Ridomil Gold.

The N90 nanoemulsion at 500 ppm appears to be a promising alternative to reduce the use of chemical fungicides in agriculture, as it significantly controlled crown and root rot disease in English walnut plants, when compared with the inoculated control treatment.

While these results are promising, further research is needed to validate these findings in commercial English walnut orchards. Conducting field trials will help to evaluate the impact of the nanoemulsion on walnut production under real-world conditions. Additionally, toxicity studies and investigations into the modes of action of these nanoemulsions are essential to ensure their safety, and to optimize their application in sustainable agriculture.

## 6. Patent

Nanoemulsions based on plant extracts were entered into the INAPI registry for intellectual protection of this technology. Chilean Patent Application No. 202003396. The registration number is 66624, under the title “Método para preparar nanoemulsiones antifitopatógenas a base de partes aéreas de *P. glansulosa* y de *E. Illinita*”.

## Data Availability

The data presented in this study are available on request from the corresponding author.
